# Feelings from the Heart Part II: Simulation and Validation of Static and Dynamic HRV Decrease-Trigger Algorithms to Detect Stress in Firefighters

**DOI:** 10.3390/s22082925

**Published:** 2022-04-11

**Authors:** Christian Rominger, Andreas R. Schwerdtfeger

**Affiliations:** Health Psychology Unit, Department of Psychology, University of Graz, 8010 Graz, Austria

**Keywords:** heart rate variability, interactive ambulatory psychophysiological assessment, simulation, stress

## Abstract

Several mobile devices have multiple sensors on board and interact with smartphones. This allows for a complex online evaluation of physiological data, important for interactive psychophysiological assessments, which targets the triggering of psychological states based on physiological data such as heart rate variability (HRV). However, algorithms designed to trigger meaningful physiological processes are rare. One exception is the concept of additional HRV reduction (AddHRVr), which aims to control for metabolic-related changes in cardiac activity. In this study we present an approach, based on data of a previous study, which allows algorithm settings to be derived that could be used to automatically trigger the assessment of psychosocial states by online-analysis of transient HRV changes in a sample of 38 firefighters. Settings of a static and a dynamic AddHRVr algorithm were systematically manipulated and quantified by binary triggers. These triggers were subjected to multilevel models predicting increases of objective stress during a period of 24 h. Effect estimates (i.e., odds) and bootstrap power simulations were calculated to inform about the most robust algorithm settings. This study delivers evidence that a dynamic AddHRVr algorithm can trigger transitions of stress, which should be further validated in future interactive psychophysiological assessments.

## 1. Introduction

The number of sensors implemented in mobile ECG devices has remarkably increased in recent years. Most available mobile ECG devices nowadays have several sensors on board and interact with smartphones. State of the art ECG devices additionally assess parameters such as movements by means of accelerometers, the sea level by means of pressure sensors, and even temperature and electrodermal activity [1]. This combination of sensors allows for a complex evaluation of physiological functioning of the autonomic nervous system and its interaction with the central nervous system [2] by assessing people’s ECG and taking corresponding movements and energy expenditure into account. However, most research uses sensor data offline, and online approaches processing data in real-time are largely missing.

Specifically, an interactive psychophysiological assessment needs (simple) online algorithms, which can identify episodes of transient bodily changes potentially signaling psychosocially relevant states in daily life. For example, Ebner-Priemer et al. developed a functional algorithm to detect episodes of intensified physical activity to trigger the assessment of wellbeing [3]. These authors only used accelerometer information for their algorithm; however, nowadays researchers strive to develop online and real-time systems to identify (conscious and subconscious) psychosocial states [4] associated with increased vulnerability and stress by using combined information of ECG and accelerometers [5,6,7,8]. This approach mainly grounds on the concept of additional heart rate [9] and additional heart rate variability reduction (AddHRVr; [6,7]), which assumes that metabolically independent HRV decreases may result from cognitive and emotional factors [10]. AddHRVr should allow conclusions about individual psychosocial states and should therefore indicate transitions from situations with lower stress to situations with elevated stress.

The AddHRVr algorithm assumes that transient HRV reductions do not only indicate metabolic needs of the organism, but are also sensitive for the complex interplay between the autonomic and central nervous system [2,11,12,13]. Specifically, the vagus nerve as the primary parasympathetic nerve and major constituent of HRV ensures a rapid communication between the brain and the heart (~200 ms) with afferent fibers (from the heart to the brain) outweighing efferent fibers (from the brain to the heart). Hence, vagally-mediated HRV could signal cognitive function, emotion regulation, and states of vulnerability and stress [2]. This is in accordance with several prominent theories, which account for the salient role of HRV for psychosocial functioning (e.g., theory of neurovisceral integration, [13]; polyvagal theory, [14]; vagal tank theory, [15]).

Specifically, the root mean square of successive differences (RMSSD) and the high frequency (HF) component of the heartbeat are indicators of HRV and primarily reflect vagal function [16,17,18]. These measures seem to be especially sensitive to higher central nervous system function and thus could be of special importance for psychosocial functioning [11,19,20,21,22]. Taken together, analyzing HRV by taking the metabolic needs into account may inform about the psychosocial functioning of an organism and may indicate transitions of stress in an ever-changing environment. However, how can we arrive at a specific AddHRVr algorithm that is sensitive to psychosocially meaningful situations in daily life, thus enabling for an interactive psychophysiological assessment?

Schwerdtfeger and Rominger presented a twostep simulation approach to derive AddHRVr algorithms adjustments that could be used to develop online algorithms for interactive psychophysiological assessments to identify periods of vulnerability and stress in everyday life [5]. In a first step, the authors assessed the individual association between HRV and bodily movement [6]. This is realized by regression analyses of the continuously recorded vagally-mediated HRV (RMSSD) on a minute-by-minute basis and the corresponding bodily movements (and associated energy expenditure). Based on this linear regression information, individual reductions of RMSSD can be estimated independent of metabolic demands. Following the algorithm, a meaningful RMSSD decrease takes place when the deviation of the momentary HRV from the predicted HRV (based on the energy expenditure) reaches a pre-defined RMSSD threshold (i.e., 0.5 SDs of the RMSSD during calibration period). The algorithm then delivers a binary trigger, indicating an AddHRVr, whenever a predefined number of meaningful RMSSD decreases (i.e., RMSSD threshold) are observed within a predefined time (i.e., RMSSD window; [5,6,7]). This individual trigger distribution simulates the online algorithm functioning and allows one to identify potentially meaningful situations. In the second step, this information can be applied to bootstrapped multilevel analyses to evaluate if a specific algorithm setting is associated with specific affective states, resilience, or stress levels, among others. Algorithms with sufficient power, acceptable effect sizes, and a feasible number of delivered triggers would be considered for future applications in online studies. Based on this approach, Schwerdtfeger and Rominger showed that, in principle, a specific setting of an AddHRVr algorithm can be specified to index specific psychosocial states (i.e., low quality of social interactions).

However, previous validation approaches were based on subjective ratings of psychological states randomly assessed during an ecological momentary assessment (EMA; [5,6,7]) and—to the authors’ knowledge—there is no simulation study available focusing on more objective measures of stress. Therefore, we used an already published data set [23], which includes 38 male firefighters who each wore a mobile ECG device for 24 h, which recorded ECG and movement associated energy expenditure. Furthermore, the operations of the firefighters were classified into three increasing levels of objective stress (i.e., routine work at the fire station, routine operations, emergency operations). The timing of these operations was based on the official operating times of the primary control unit (for a similar procedure, see, e.g., [8]). This allowed us to estimate (1) when a transition of objective stressfulness occurred, and (2) whether it was an increase (e.g., from routine operations to emergency operations) or a decrease of objective stressfulness (e.g., from emergency operations to routine work at the fire station). Furthermore, since all available studies on the AddHRVr algorithm focused on static algorithms, we additionally simulated a dynamic algorithm in this study [5,7]. In contrast to a static AddHRVr algorithm, a dynamic algorithm is specifically designed to adapt to participants’ HRV deviations, which might further increase the sensitivity to detect AddHRVr due to transitions of objective stress.

Therefore, this simulation study examined if a specific setting of an algorithm (static or dynamic) could identify an increase of objective stressfulness, while leaving decreases of stress largely undetected. We were further interested whether a dynamic algorithm might outperform a static algorithm or not. Hence, this study aims to provide further evidence for the validity of algorithms to detect meaningful stress-related decreases of HRV independently from metabolic demands.

## 2. Method

### 2.1. Participants

An already published data set of Schwerdtfeger and Dick was used to simulate the algorithm settings [23]. In total, 38 male firefighters took part in this study. The mean age of the participants was *M* = 32.71 years (*SD* = 6.90). An EMA was conducted to collect data throughout 24 h (for details see [23]). The study was approved by the ethics committee, and informed consent was obtained from all participants.

### 2.2. Material and Instrument

#### 2.2.1. EMA

At each random and self-paced prompt, the firefighters rated their perceived stress with two items (‘I feel stressed’, ‘I feel burdened’). Furthermore, state negative affect was assessed with five items from the positive and negative affect schedule (PANAS, [24]) with the following items: ‘I am upset’, ‘I feel distressed’, ‘I feel agitated’, ‘I feel tense’, ‘I am nervous’ (for more details see [23]). In total, 571 valid prompts were available, which took place during one of the three objective situations of increasing stress. Between-person (R_kR_) and within-person (R_C_) reliability was good for both measures (stress: R_kR_ = 0.87, R_C_ = 0.80; negative affect: R_kR_ = 0.94, R_C_ = 0.71).

#### 2.2.2. Objective Changes of Stress: From Routine Work at the Fire Station to More Stressful Emergency Operations

Work episodes were continuously coded (in 1-min steps) as either covering routine work at the fire station (non-stressful, 81.4% of the 24 h), low-stressful routine operations (11.2% of the 24 h), or high-stressful emergency operations (7.4% of the 24 h). There were 199 operations in total (*M* = 5.24 per participant) of which 40% were coded as highly stressful (see [23] for more details). For the present study, we calculated the moment when a change in objective stressfulness took place, and the firefighters had a routine or emergency operation. The timing of the three levels of stress was based on the official operating times of the primary control unit. Based on this continuous information (1-min steps), we identified moments when an increase of objective stressfulness was observed (i.e., a change from non-stressful to low-stressful, from non-stressful to high-stressful, and from low-stressful to high-stressful operations). Furthermore, we identified the moments when a decrease of objective stressfulness took place. We only considered objective changes in stressfulness when these situations lasted at least 20 min. Moments of objective stress increases were coded with 1 (*n* = 182), and moments of decreases of objective stressfulness were coded with 0 (*n* = 170; e.g., from high-stressful to low-stressful operations). The mean time between changes of stress was 116.18 min (*SD* = 142.34 min) with a minimum of 20 min and a maximum of 909 min. Based on this information, we were able to calculate if AddHRVr triggers were associated with moments of increases or decreases of objective stress. We expected that especially increases in objective stress should go along with AddHRVr triggers, and a decrease should go along with the absence of AddHRVr triggers. Therefore, we expected a positive relationship at the second step of analyses.

#### 2.2.3. Physiological Ambulatory Monitoring of ECG and Movement

ECG and bodily movement were recorded with the physiological ambulatory monitoring device EcgMove3 (movisens GmbH, Karlsruhe, Germany) throughout one weekday (24 h). The ECG signal was sampled with 12 bit-resolution and stored with 1024 Hz. Bodily movement was recorded with 64 Hz via a 3D acceleration sampling. In combination with an integrated pressure sensor, activity energy expenditure (AEE) in kcal was calculated.

### 2.3. Data Preprocessing

The EcgMove3 device delivers information of several variables including HRV, movement, and AEE in real time. The device calculates relevant variables (e.g., RMSSD) in adjacent 1-min segments, which could be used for the online application of an algorithm. We used the stored live RMSSD data of the device for the simulation of the algorithm function. These stored online values are automatically scanned for artifacts by the movisens EcgMove3 device during recording. We used the established time domain measure RMSSD (ms) to assess HRV and AEE (kcal) to assess metabolic changes due to movement.

### 2.4. Simulation of a Dynamic and a Static Algorithm for Detecting AddHRVr

In this work, we applied the two-step approach of simulating and developing an algorithm to work in online mode presented by Schwerdtfeger and Rominger [5]. In step 1, the AddHRVr algorithms were simulated at the individual level. By simulating various algorithm adjustments separately for a static and a dynamic algorithm, it can be determined when an algorithm would have detected meaningful HRV decreases and delivered triggers within the 24 h of recording. In step 2, these triggers were used to predict the objective increase of stress. By running bootstrapped multi-level analyses per algorithm setting (500 iterations each), predicting the increase of stress based on the association with a trigger (within 20 min after the objective change of stress), the power and the mean odds associated with a specific algorithm setting were calculated [5].

#### 2.4.1. Step 1: Simulation of Individual AddHRVr Triggers for Each Firefighter

As outlined by Schwerdtfeger and Rominger, the association between AEE and HRV differs between persons [5]. Therefore, a linear regression analysis predicting each firefighter’s RMSSD (ms) by AEE (kacl) was calculated at the first step. This is necessary for the calibration of the algorithm and to account for metabolic demands [5,7]. Individual linear regressions were based on the total 24 h of recorded data [25]. The resulting scatter plots and linear regression lines were visually inspected for each participant to indicate if outliers were present. Few 1-min segments were automatically deleted before calculating regression analyses (*M* = 0.16, *SD* = 0.50, max = 2; for further methodological details see [5]).

**Figure 1:**
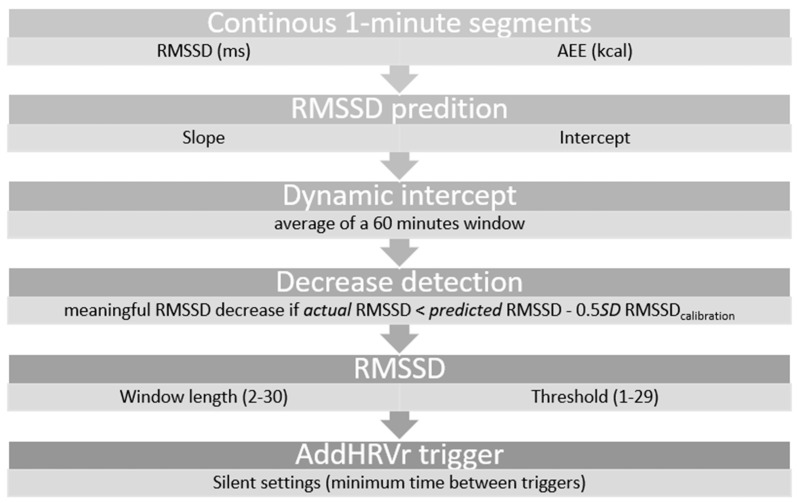
An adapted schematic representation of a dynamic AddHRVr algorithm from Schwerdtfeger and Rominger [5].

The individual linear regression parameters (i.e., intercept and slope) were then used to simulate the algorithms and calculate meaningful RMSSD decreases (see Figure 1). For the static algorithm, the continuous 1-min AEE scores were used to calculate the expected RMSSD (due to the regression function), which was compared with the corresponding and actual RMSSD of this very minute. If the deviation between actual RMSSD and predicted/expected RMSSD was higher than a predefined threshold (i.e., 0.5 × SD of RMSSD_calibration_; see Table 1), this 1-min segment was classified as a meaningful RMSSD decrease.

**Figure 2:**
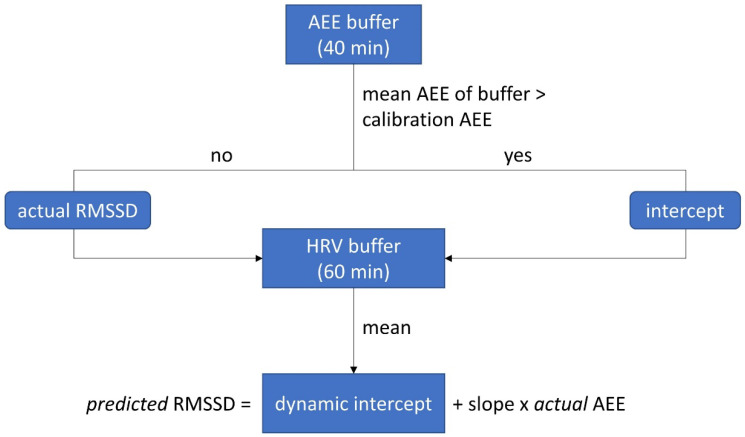
Decision tree of the dynamic intercept implemented in the dynamic AddHRVr algorithm.

Since the dynamic algorithm should account for (psychologically relevant and) dynamic changes of HRV during the day and therefore should adapt to different HRV levels, we applied a moving average procedure to the continuously recorded HRV signal. The mean HRV (RMSSD) of a 60-min buffer serves as the dynamic intercept to predict the expected RMSSD of each single minute (see Figure 2). The content of this buffer changes in 1-min steps, which allows a continuous algorithm adjustment for each minute. The buffer is filled with the corresponding HRV value (of the very minute) if the observed mean AEE of the last 40 min is lower than the average AEE during the calibration. If the observed mean AEE of the last 40 min was higher than the average AEE during calibration, the 60-min buffer is filled with the intercept derived from the linear regression analysis (i.e., HRV without metabolic demands; for the decision tree see Figure 2). This replacement of HRV values is necessary, since HRV values accompanied with high AEE will most likely be influenced by movement and corresponding metabolic demands and might therefore not adequately indicate the intended (psychologically relevant and) dynamic HRV changes during the day. The algorithm starts with an HRV buffer with the intercept as average and an AEE buffer with the mean AEE during calibration as average.

According to the algorithm, a 1-min segment classified as a meaningful RMSSD decrease is not sufficient to provoke an AddHRVr trigger. As illustrated in Figure 1, three further parameters are implemented in the algorithms: (1) the RMSSD window length (number of 1-min segments included), (2) the RMSSD window threshold (the number of 1-min segments, which have to be classified as a meaningful RMSSD decreases in order to provoke an AddHRVr trigger), and (3) the silent setting. Specifically, if within a predefined period of 5 min (i.e., window length), 4 segments are classified as significant decreases (i.e., RMSSD window threshold), an AddHRVr trigger will be provoked (e.g., 4 out of 5). Following an AddHRVr trigger, the algorithm will remain silent for a predefined time (i.e., silent setting, e.g., 20 min), which prevents the algorithm to trigger further prompts.

Importantly, the change of these parameters significantly alters the characteristic of the algorithm (for a detailed exploration of a static algorithm, see [5]). For example, an algorithm which fires when 4 out of 5 segments are classified as meaningful HRV decreases detects predominantly shorter-lived effects as compared to an algorithm with a 7 out of 10 or even a 13 out of 30 setting. Hence, different algorithms are associated with different alarm-rates and might differ in their psychosocial meaningfulness. For reasons of parsimony, we followed Schwerdtfeger and Rominger and mainly focused on the window length and window threshold and kept the silent setting of 20 min constant [5]. We calculated the resulting trigger information (coded as 0 = absent and 1 = present) at the individual level for all combinations of RMSSD window lengths starting from 2 to 30 and RMSSD window thresholds from 1 to 29 (i.e., 1 out of 2 until 29 out of 30; i.e., 435 different algorithm adjustments). These 435 different trigger distributions were the input for the multi-level simulation at step 2.

#### 2.4.2. Step 2: Simulation of the AddHRVr Triggers to Predict Objective Changes of Stressfulness

Similar to former procedures [5,7,25], the predictive value of an AddHRVr trigger relative to an increase of objective stress was determined via calculating the associations of an AddHRVr trigger within the transition of objective stress. Thus, we aimed to evaluate the sensitivity of various AddHRVr algorithms by comparing the associations of AddHRVr triggers with the objective change of stressfulness (i.e., increase vs. decrease of stress). A reliable association between transitions of objective stress and AddHRVr triggered prompts would suggest psychophysiological sensitivity of the algorithm settings. Statistical evaluation was accomplished via the lme4 package (linear mixed effects modeling [26]) in R (version 4.0.4 [27]) using the glmer function (generalized linear mixed-effects models).

Specifically, within 20 min after an objective change of stressfulness, the prevalence of an AddHRVr trigger was determined. The triggers identified (coded as 0 = absent and 1 = present) were subjected to a multilevel model predicting increases of objective stressfulness. In total, 435 different combinations of trigger settings were analyzed (i.e., RMSSD window length, RMSSD window threshold) with a silent setting of 20 min. These 435 multilevel models were bootstrapped with 500 iterations each. For each iteration, data of 38 participants were sampled with replacement. We estimated statistical power, effect sizes (i.e., odds), confidence intervals, and the mean number of triggered increases and decreases of all combinations of the algorithm’s settings. Statistical power was calculated by dividing the number of iterations with a *p* < 0.05 by the total number of (valid) iterations (hence, the ratio between significant effects and total iterations). Based on this information, 3-dimensional hyperplanes were generated in R (plotly package [28]) to visualize the properties (i.e., power) of the different algorithm settings (i.e., window length and threshold). In accordance with Schwerdtfeger and Rominger, an algorithm setting with high power, solid effect size (confidence intervals), and a reasonable number of AddHRVr triggers should be favored for an online validation study [5].

## 3. Results

### 3.1. Perceived Stress, Negative Affect, and HRV (RMSSD) during the Three Different Levels of Objective Stress (Routine Work vs. Routine Operations vs. Emergency Operations)

In order to provide evidence for validity of the objective levels of stress, we calculated three random intercept models with the objective level of stress as fixed effect predicting perceived stress, negative affect, and HRV (mean RMSSD 10 min before each prompt). These three analyses indicated increased stress and negative affect during routine operations (vs. routine work at the fire station; see Table 1) and during emergency operations (vs. routine work at the fire station). In accordance with this, HRV (RMSSD) showed decreases in these situations, which were independent from changes in AEE. As an important prerequisite to simulate AddHRVr algorithms to detect objective changes in stress, this pattern of findings provides evidence for the validity of the objective classification of stressfulness in firefighters.

### 3.2. Simulation of Static and Dynamic AddHRV Algorithms

#### 3.2.1. Step 1: AddHRVr Algorithm Simulation on an Individual Level

Table 2 presents the descriptive statistics of the resulting individually adjusted parameters of the static and dynamic algorithms by means of a linear regression approach. All parameters showed high interindividual variation.

**Figure 3:**
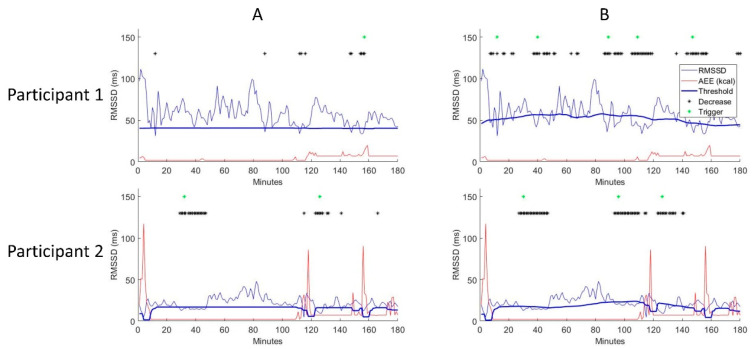
Example of a simulation for two firefighters. Both algorithms were run with a window threshold of 4 and a window length of 6 (i.e., 4 out of 6) with a silent period of 20 min in between. The figure illustrates an observation time of 3 h. The *x*-axis depicts minutes and the *y*-axis RMSSD. The red line represents the amount of AEE (kcal), the blue line is the actual RMSSD, and the bold blue line represents the estimated threshold (predicted RMSSD—0.5 × SD RMSSD_calibration_). Green asterisks indicate AddHRVr triggers, and the black asterisks indicate a 1-min segment with the actual HRV being lower than the predicted threshold. Panel (**A**) represents a static and Panel (**B**) a dynamic AddHRVr algorithm.

Based on this information, the distribution of static and dynamic AddHRVr triggers can be simulated individually. Panel A of Figure 3 shows the AddHRVr triggers for a dynamic algorithm setting and panel B for a static algorithm (both with 4 out of 6). The number as well as the temporal distribution of triggers (green asterisks) substantially differed between the static and the dynamic algorithm. This difference of delivered triggers can be explained by the intended properties of the dynamic algorithm, which adapts to changes of participant’s HRV levels. These adaptations result in a dynamic change of the estimated threshold (predicted RMSSD—0.5 × SD RMSSD_calibration_; bold blue line in Figure 3), which allows one to detect meaningful decreases of HRV even if the level of HRV increased.

**Figure 4:**
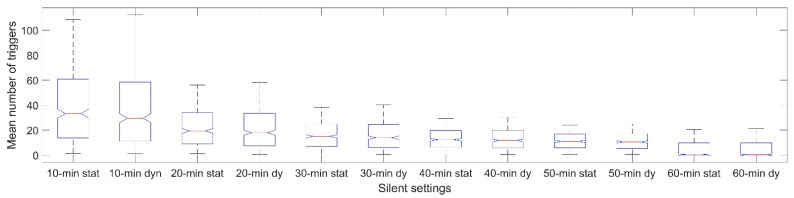
Mean number of AddHRVr triggers for 6 different silent settings of the algorithm (from 10 to 60 min) for the static and the dynamic algorithm, separately.

Furthermore, as illustrated in Figure 4, the dynamic algorithm was associated with a lower total number of delivered triggers in contrast to the static algorithm when the silent setting was set to 10 min (*t*(434) = 27.82, *p* < 0.001), 20 min (*t*(434) = 19.60, *p* < 0.001), 30 min (*t*(434) = 11.98, *p* < 0.001), and 40 min (*t*(434) = 6.11, *p* < 0.001), but was not significantly different with a silent setting of 50 min (*t*(434) = 0.14, *p =* 0.892). The dynamic algorithm was associated with a higher total number of delivered triggers, when the silent setting was 60 min (*t*(434) = −5.71, *p* < 0.001). For a silent setting of 20 min, which was applied in the present simulation, the mean total number of delivered static triggers per setting was *M* = 22.14 (*SD* = 15.09) and for the dynamic algorithm *M* = 21.31 (*SD* = 15.73).

#### 3.2.2. Step 2: Simulation of Algorithm Settings to Detect Objective Transitions of Stress

**Figure 5:**
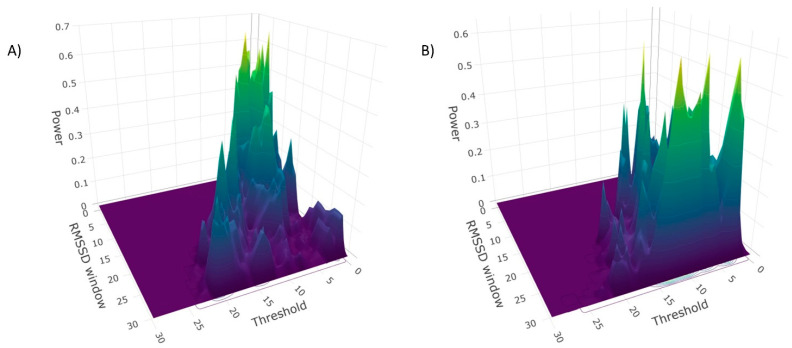
Left panel (**A**) illustrates the power for each of the 435 bootstrapped multi-level analyses using the dynamic algorithm settings of RMSSD window length (*x*-axis) and window threshold (*y*-axis; i.e., y out of x to be a trigger; see [29] for an interactive 3D illustration). Panel (**B**) illustrates the power for each of the 435 bootstrapped multi-level analyses using the static algorithm (500 samples with *n* = 38; for an interactive 3D illustration see [30]). The silent setting of both figures was 20 min.

In order to derive the most sensitive algorithm setting for predicting an increase of stress, all 435 bootstrap simulations were inspected for the highest power separately for the static and the dynamic algorithm (i.e., a total of 870 bootstrapped simulations; Figure 5A; see [29] for an interactive 3D illustration of the dynamic algorithm). The highest power of 0.680 was observed for the algorithm setting with 7 out of 10 (silent setting of 20 min). Table 3 and Table 4 shows the adjustments with similar power scores for the dynamic and the static algorithm. Effect estimates are the percentage change in odds of being an increase of objective stress (i.e., odds ratio−1) × 100). This means that when within a time window of 20 min after a transition of stress, a trigger was delivered, this increased the odds of being an increase of objective stress by, e.g., 99% in the case of the algorithm setting with 7 out of 10 (see Table 3).

Although the power scores were not significantly different between the dynamic and the static AddHRVr algorithm (*t*(427) = 0.36, *p* = 0.722), the observed estimated effects for the dynamic algorithm were significantly more positive as compared to the effects of the static algorithm (*t*(427) = 6.09, *p* < 0.001). When additionally taking the specific algorithm adjustments into account, it could be concluded that the dynamic AddHRVr algorithm predicted increases of objective stress more sensitively compared to the static algorithm (Table 3 and Table 4). Specifically, if within a time window of 20 min after a transition of stress, a trigger was delivered, this increased the odds of being an increase of objective stress by 99% at an algorithm setting of 7 out of 10. The total number of delivered triggers in this setting was 578, thus indicating that each participant would have received about 15.21 triggers within the 24 h of recording in case an interactive psychophysiological ambulatory assessment would have been conducted with these settings.

For the static algorithm, a setting 13 out of 30 showed the highest power of 0.624 (see Table 4 and Figure 5). However, the estimated effect size was negative, thus suggesting that within a time window of 20 min after an objective transition of stress, a delivered trigger would decrease the odds of being an increase of stress by 44%, and the algorithm triggered more decreases of stress (i.e., 50.30) compared to increases (i.e., 35.27; see Table 4).

**Figure 6:**
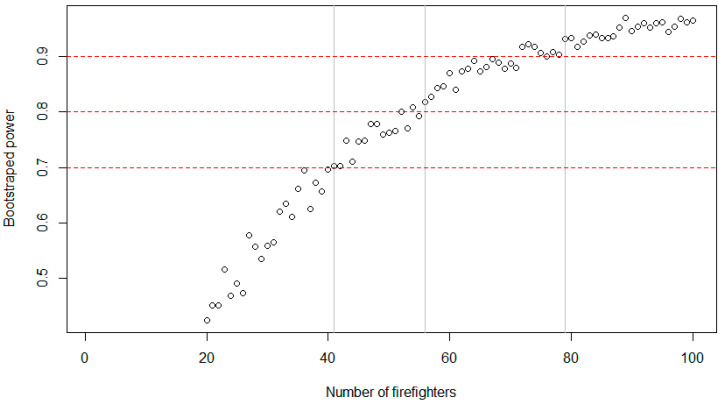
Illustration of bootstrap simulations (500 samples each) of the association between power (*y*-axis) of the 7 out of 10 setting (20 min silent setting) and the sample size (*x*-axis). Red dashed horizontal lines represent the power thresholds of 0.70, 0.80, and 0.90. The grey solid vertical lines represent the number of firefighters at least needed to reach a robust power of 0.70, 0.80, and 0.90.

Since the achieved power of the dynamic algorithm did not reach the 0.70 threshold, we further simulated how many participants should be sampled in an online study to reach a sufficient power with the suggested setting of 7 out of 10 (silent setting 20 min). As depicted in Figure 6, the simulation reached a robust power of above 0.70 with a samples size of *N* = 41, the 0.80 threshold with *N* = 56 participants, and a power of 0.90 with *N* = 79 participants.

## 4. Discussion

The aim of this study was to demonstrate a simulation approach to derive the settings of a static and a dynamic AddHRVr algorithm to index increases of stress. We were specifically interested to show that this simulation approach can be applied to objective indicators of transitions of stress in firefighters, indicating the validity of an AddHRVr algorithm. By simulating algorithm settings along several dimensions, separately for a static and a dynamic algorithm, we arrived at an algorithm specification of 7 out of 10 min-segments with AddHRVr exceeding an individually predefined threshold of predicted RMSSD for a dynamic algorithm. Importantly, this study applied a procedure that could be useful to derive sensitive settings for a psychosocially meaningful AddHRVr algorithm. While previous research was mainly concerned with static algorithms [6,25] and focused on subjective psychological states [5], we applied an explorative approach to determine which algorithm settings are particularly sensitive to objective transitions of stressfulness in firefighters, which in turn are associated with decreased HRV (RMSSD) as well as increased perceived stress and subjectively rated negative affect [8,31].

It should be noted though that the derived settings in this study at step 2 could differ in other populations and particularly for other psychosocial concepts (e.g., worry, rumination, anger, or fear). However, the individual parameters derived at step 1 of the present study are similar to parameters calculated by Schwerdtfeger and Rominger, although they predominantly investigated young students [5]. Specifically, the observed RMSSD and the linear correlation between bodily movement and RMSSD are largely comparable. This finding indicates some robustness of the linear regression approach for individual algorithm adjustments based on physiological ambulatory assessment of several hours during everyday life [5,25]. Nonetheless, it seems mandatory to validate the findings of step 2 in subsequent research and to analyze the specificity of the algorithm settings (for further details on the validation of derived algorithms see [5]). Finally, online application of the derived algorithm settings is the gold standard of validation.

However, a simulation approach is essential to come up with algorithm settings that would work online, since the potential settings of an algorithm are infinite. Doing this in the field by applying different settings in online studies in different samples would certainly not be feasible. To systematically apply different settings of RMSSD window length and the RMSSD threshold in online studies, we would have needed 435 different samples with 38 firefighters each. This would have resulted in a total sample size of 15,530 firefighters. For a systematic evaluation in a within-subjects design, we would have needed the observation of 38 firefighters for 435 days with changing settings each 24 h. Additionally, the sampling needs would be further multiplied if researchers are interested in the impact of variations of the silent settings or are interested in comparing static and dynamic AddHRVr algorithms.

The dynamic AddHRVr algorithm, adapting for previous HRV (60 min), constitutes a promising alternative to a static algorithm. The present simulation approach indicated that a dynamic AddHRVr algorithm shows remarkably different characteristics compared to a static algorithm. First, the number of delivered triggers was significantly lower for various silent settings (from 10 min to 40 min). Second, the power analysis derived from the bootstrap method showed a different pattern of peaking regions (although there was no mean difference of power). While the dynamic AddHRVr algorithm showed the highest power at more shorter-lived settings (e.g., 7 out of 10), the static algorithms were based on longer RMSSD windows lengths (e.g., 13 out of 30). Third, the observed effects were more positive for the dynamic algorithm compared to the static algorithm. A closer look at the most powerful settings indicated that the dynamic algorithm showed the expected positive effects, and the static algorithm showed even negative effects and a reversed pattern of delivered triggers.

This is an astounding result and indicates the high complexity of algorithm settings, since positive odds were expected for both the static and the dynamic algorithm. However, negative effect sizes argue for the assumption that some of the simulated static AddHRVr algorithm settings are not valid and therefore not detecting stress. This interpretation is in line with the observation that the most powerful setting of the static algorithm was 0.624 (i.e., 13 out of 30), which was relatively low. Furthermore, the power illustrations of the static and dynamic algorithms presented in Figure 5 indicate that the pattern of peaking for the static algorithm seems to be less localized compared to the dynamic algorithm. These observations are in line with the assumption that the static algorithm might deliver invalid triggers not associated with increases of stress and therefore more likely assess other psychological aspects of HRV reductions. Furthermore, it should be noted that the simulation is based on the assumption that increases of objective stress should be triggered within 20 min following the beginning of an operation. Therefore, the simulation approach also includes rapid psychophysiological fluctuations, for which the dynamic AddHRVr algorithm seems to be more sensitive. This assumption is further strengthened by the observation that the static algorithm only achieved a power of 0.424 with an odd of 69% for the setting 7 out of 10 (which was the most powerful setting for the dynamic algorithm). However, when focusing on longer effects (within 40 min) and longer operations (at least 40 min), the static algorithms showed better power scores. The setting of 1 out of 10 reached even a power of 0.980 with percentage change in odds of 365% (with a silent setting of 20 min; for a 3D illustration of power see [32]; for odds see [33]). However, this setting would deliver 46.61 triggers within 24 h per participant, which would not be applicable in psychophysiological assessment studies.

Furthermore, it should be noted that in addition to a static linear AddHRVr algorithm [5], also a static inverse AddHRVr algorithm was reported in the literature [6,7]. The inverse algorithm assumes a linear association between HRV and the inverse of bodily movement. Correspondingly, the intercept in a static inverse approach means HRV at very high levels of bodily movement (i.e., infinite), while in a static linear regression approach, the intercept represents the participants’ HRV without movement, which allows its continuous replacement with the measured HRV (at low levels of bodily movement). Therefore, the dynamic algorithm cannot easily be transferred to the inverse approach, hampering a direct comparison in this study. Nevertheless, we additionally simulated the settings of an inverse AddHRVr algorithm by means of the available data. This simulation indicated good power scores with 0.804 (3D illustration [34]) as well as percentage change in odds of 97% for the setting 6 out of 12 (3D illustration [35]). Furthermore, this setting of the inverse algorithm was associated with 19.58 triggers within 24 h. This indicates that a static inverse AddHRVr algorithm can outperform a static linear algorithm, and that the performance outcome of the inverse algorithm is largely comparable with the performance of the dynamic approach, thus ultimately underlining the validity of the dynamic AddHRVr algorithm to detect transitions of stress.

In contrast to previous studies, we indicated that dynamic (and the inverse) AddHRVr algorithms can predict objective changes in stressfulness in a sample of firefighters quite early (within 20 min) accompanied by increases of stress and negative affect [8]. The dynamic algorithm settings can detect these situations, thus arguing for the application of interactive psychophysiological ambulatory assessments to unobtrusively detect situations of interest (i.e., stressful moments in an individual’s life). At this point of argumentation, some might mention that the odds to detect increases of objective stressfulness did not reach high levels as might be achieved by other methods, such as deep learning networks and artificial intelligence approaches [8,36,37], and increasing the sample size and using data-driven approaches (e.g., machine learning, compressed deep learning) could be an alternative approach for the present study. It should be held in mind, however, that firstly, these machine learning approaches do not work online [8]. However, compressed deep learning networks to classify heartbeats and arrythmia were recently developed [38,39]. Hence, compressed deep learning networks to detect stress in everyday life could constitute promising tools in ambulatory research in the future. Secondly, although the present study was concerned with the detection of objective transitions of stress for validation purposes, the focus of the AddHRVr algorithm is to trigger psychologically meaningful increases of stress and negative affective states (i.e., reduced resilience) independent of metabolic demands, which might also occur during routine operations (similar argumentation, see [8]). Thirdly, the AddHRVr algorithm is a top-down and theory driven approach, which contrasts with data driven and bottom-up machine learning approaches. Alternative algorithms solely taking data from the acceleration sensor into account [3] might provide even better classifications of transitions of objective stress, since leaving the fire station (and showing strong increases in physical activity) is strongly associated with routine and emergency operations and therefore increases of objective stress (see, e.g., [8], thus indicating improved classification of emergency episodes in firefighters by additionally analyzing acceleration sensor data). The main aim of this study, however, was to demonstrate that an AddHRVr algorithm can systematically trigger situations which are associated with increased stress and negative affect independent of metabolic demands and bodily movements and did not directly target the issue of (external) validity, where the relatively small sample size of the current study might have been an issue. This is in some contrast to other research in this field and has to take more than the observed sensitivity into account. In addition to the power, also the (direction of) effect size and the number of delivered triggers are essential parameters. Researchers should decide about potential online applications of specific algorithm setting in future field studies after careful consideration of these parameters. Therefore, this simulation study provides additional evidence for theory-driven psychophysiological assessment in daily life. Fourth, in contrast to most bottom-up methods, the applied algorithm did not use all available information at once but works sequential, which allows online application. Nevertheless, future simulation studies should attempt to further increase the sensitivity of the AddHRVr algorithm function to detect situations of increased stress by means of static (linear and inverse) as well as dynamic algorithm approaches.

On a final note, it is important to keep in mind that not only stress is associated with decreased HRV [40], but also perseverative cognition, worry, and rumination [7,41,42,43,44]), anxiety [19], depression [45,46,47], lower quality of interactions [5], and even activated/arousal-related positive (motivational) states assessed in everyday life [23,48]. This nicely outlines the potential applications of static and dynamic algorithms in future ambulatory research. When comparing the present findings with the simulation study of Schwerdtfeger and Rominger, it seems likely that different phenomena might be associated with different patterns of (momentary) HRV reductions [5]. This further increases the need for further simulation studies to come up with algorithm settings for static and dynamic AddHRVr algorithms allowing one to trigger different psychologically meaningful situations in everyday life.

## 5. Conclusions

Schwerdtfeger and Rominger concluded that we can probably detect meaningful psychosocial episodes by an online analysis of HRV enabled by ECG devices that have several sensors on board [5]. However, this search of a needle in a haystack needs considerable methodological effort and simulations of various AddHRVr algorithms in different samples assessing various indicators of stress (i.e., subjective and objective), affect, and resilience. This study of firefighters adds evidence to this line of research and suggests that dynamic (and inverse!) AddHRVr algorithms could detect objective transitions of stress that are associated with higher levels of perceived stress and negative affect. Therefore, this study contributes to the development of an interactive psychophysiological ambulatory assessment approach and argues for the assumption that several algorithm adjustments might exist that show similar properties to trigger psychologically meaningful episodes in our daily lives.

## Figures and Tables

**Table 1:** Multilevel model for objective stress predicting perceived stress, negative affect, and HRV (RMSSD).

Parameter	Estimate (SE)	df	t	*p*
Model 1: Perceived Stress				
Intercept	1.32 (0.06)	531	21.17	<0.001
routine operations (vs. work at the fire station)	0.54 (0.07)	531	7.60	<0.001
emergency operations (vs. work at the fire station)	1.04 (0.08)	531	12.79	<0.001
Model 2: Negative affect				
Intercept	1.31 (0.05)	531	23.90	<0.001
routine operations (vs. work at the fire station)	0.20 (0.05)	531	4.11	<0.001
emergency operations (vs. work at the fire station)	0.46 (0.06)	531	8.31	<0.001
Model 3: HRV (RMSSD)				
Intercept	45.17 (2.87)	524	15.73	<0.001
routine operations (vs. work at the fire station)	−3.85 (1.55)	524	−2.49	0.013
emergency operations (vs. work at the fire station)	−5.01 (1.79)	524	−2.81	0.005
AEE (kcal)/10^3^	−5.18 (0.67)	524	−7.71	<0.001

**Table 2:** AddHRVr algorithm calibration: Descriptive statistics of the individual parameters for all 38 firefighters calculated for 24 h of recording.

	*M*	*SD*	*Max*	*Min*
RMSSD (ms)	45.20	20.63	96.18	18.40
AEE (kcal)	1026.48	184.41	1428.89	680.78
Intercept	51.05	24.04	106.21	19.37
Slope (per 1000 kcal)	−5.96	4.46	−0.42	−17.10
*r*	−0.36	0.10	−0.06	−0.54

**Table 3:** Order of dynamic algorithm settings with respect to power estimates.

Order	Window Threshold	Power	Effect Estimate ^a^	CI Low (2.5%)	CI High (97.5%)	Total Triggers	Triggered Increases/Decreases
1	10/7	0.680	99.18%	12.81	287.02	578	46.24/25.06
2	7/1	0.662	73.40%	9.60	171.37	1706	140.21/111.90
3	11/5	0.622	68.03%	1.17	196.60	915	76.18/51.08
4	5/1	0.606	68.27%	14.41	154.56	1643	136.10/108.92

Note. ^a^ = percentage change in odds; total triggers = number of triggers delivered at the specific algorithm settings, triggered increases/decreases = number of triggered increases and decreases, i.e., trigger within 20 min after transitions of stress, total number of transitions = 352 (182 increases and 170 decreases).

**Table 4:** Order of static algorithm settings with respect to power estimates.

Order	Window Threshold	Power	Effect Estimate ^a^	CI Low (2.5%)	CI High (97.5%)	Total Triggers	Triggered Increases/Decreases
1	30/13	0.624	−43.52%	−66.47	−7.40	736	35.27/50.30
2	19/12	0.620	−54.58%	−78.28	−6.30	474	14.81/27.18
3	27/7	0.608	−37.55%	−57.59	−7.63	1222	82.82/96.82
4	28/3	0.594	−42.26%	−62.22	−8.95	1775	128.99/136.98

Note. ^a^ = percentage change in odds; total triggers = number of triggers delivered at the specific algorithm settings, triggered increases/decreases = number of triggered increases and decreases, i.e., trigger within 20 min after transitions of stress, total number of transitions = 352 (182 increases and 170 decreases).

## Data Availability

All supporting data, scripts, and figures are available under https://osf.io/5386j/ (accessed on 27 December 2021).
